# Identifying Progression-Specific Alzheimer’s Subtypes Using Multimodal Transformer

**DOI:** 10.3390/jpm14040421

**Published:** 2024-04-15

**Authors:** Diego Machado Reyes, Hanqing Chao, Juergen Hahn, Li Shen, Pingkun Yan, for the Alzheimer’s Disease Neuroimaging Initiative

**Affiliations:** 1 Department of Biomedical Engineering, Center for Biotechnology and Interdisciplinary Studies, Rensselaer Polytechnic Institute, Troy, NY 12180, USA; machad@rpi.edu (D.M.R.); chaoh.rpi@gmail.com (H.C.); hahnj@rpi.edu (J.H.); 2 Department of Biostatistics, Epidemiology and Informatics, Perelman School of Medicine, University of Pennsylvania, Philadelphia, PA 19104, USA; li.shen@pennmedicine.upenn.edu

**Keywords:** disease subtyping, artificial intelligence, multimodal biomarker, transformer network, Alzheimer’s disease

## Abstract

Alzheimer’s disease (AD) is the most prevalent neurodegenerative disease, yet its current treatments are limited to stopping disease progression. Moreover, the effectiveness of these treatments remains uncertain due to the heterogeneity of the disease. Therefore, it is essential to identify disease subtypes at a very early stage. Current data-driven approaches can be used to classify subtypes during later stages of AD or related disorders, but making predictions in the asymptomatic or prodromal stage is challenging. Furthermore, the classifications of most existing models lack explainability, and these models rely solely on a single modality for assessment, limiting the scope of their analysis. Thus, we propose a multimodal framework that utilizes early-stage indicators, including imaging, genetics, and clinical assessments, to classify AD patients into progression-specific subtypes at an early stage. In our framework, we introduce a tri-modal co-attention mechanism (Tri-COAT) to explicitly capture cross-modal feature associations. Data from the Alzheimer’s Disease Neuroimaging Initiative (ADNI) (slow progressing = 177, intermediate = 302, and fast = 15) were used to train and evaluate Tri-COAT using a 10-fold stratified cross-testing approach. Our proposed model outperforms baseline models and sheds light on essential associations across multimodal features supported by known biological mechanisms. The multimodal design behind Tri-COAT allows it to achieve the highest classification area under the receiver operating characteristic curve while simultaneously providing interpretability to the model predictions through the co-attention mechanism.

## 1. Introduction

Alzheimer’s disease (AD) is the most prevalent neurodegenerative disorder, affecting over 6.5 million people in the US alone, and its rate is expected to keep increasing [1]. Current therapies for AD are mainly focused on the management of symptoms and promising drugs that can slow the progression of the disease [2,3,4]. Therefore, early diagnosis of neurodegenerative diseases is crucial. However, early diagnosis of AD presents a significant challenge since memory loss only develops in the MCI stage of the AD continuum and cognitive decline can vary among patients due to the disease’s heterogeneity [5]. Therefore, it is crucial to develop methods capable of characterizing the factors that influence disease progression and identifying individual patients with progression-specific subtypes at an early stage.

AD is traditionally diagnosed based on characteristic cognitive decline and behavioral deficits that do not become apparent until intermediate to late stages of the disease. More recently, early-stage indicators such as imaging-based and fluid biomarkers have shown great potential for early detection of AD [6]. Fluid biomarkers found in blood and CSF have now become standard methods of diagnosing early AD patients [7,8,9], even showing great potential for subtyping AD [10]. Similarly, recent imaging-based approaches such as brain connectivity analysis in the form of connectomes have shown promising results for early diagnosis [11]. AD subtypes have been previously identified based on hallmark AD biomarkers obtained from brain imaging such as beta-amyloid [12] and tau accumulation [13,14].

Data-driven approaches have focused on classifying patients according to subtypes based on disease progression from mild cognitive impairment (MCI, a prodromal stage of AD) to AD conversion [15,16,17,18,19]. Current methods for subtyping AD and related disorders have focused mainly on using longitudinal data from clinical assessments for unsupervised learning [20,21,22]. For clustering approaches such as in [15,17,20,22], the authors subtyped AD patients using single modalities at baseline, such as blood markers [17], genomic data [20], or traits derived from imaging from longitudinal measurements [22]. These single-modality approaches using baseline data have shown the potential of early-stage indicators for AD subtyping. However, most of them fail to show how they relate biologically to AD development or use multiple time points, which hinders the ability to diagnose patients at early stages after AD onset.

Deep learning models have effectively identified diagnostic groups [23] and subtypes [18] using multimodal imaging data and correlation-based approaches that allow a greater explainability of of the relationships between the features learned by the model. Nevertheless, these are limited by two key factors, namely, the use of only imaging data and the fact that correlation-based approaches treat the clustering goal indirectly. Works on related disorders have shown the relevance of multimodal approaches [24] using non-negative matrix factorization and Gaussian mixture models or employing autoencoders and long short-term memory (LSTM) networks to learn deep embeddings of disease progression [25]. However, this requires longitudinal data that involve several time points. While several clustering and deep learning-based approaches have high accuracy when having multiple time points, the performance decreases significantly given only baseline data. This is driven by the subtle expression of the symptoms tracked in the clinical assessments limiting the scope of the models. Early-stage indicators such as imaging traits or genomic risk factors are rarely used and are simply aggregated to clinical assessments as additional indicators. Therefore, it is essential to target early-stage indicators such as genetics, imaging, and cognitive assessments.

Multimodal deep learning approaches can combine different modalities to provide a much more informed picture of disease drivers and aid in disease subtyping [26]. However, it is not a trivial task to identify the relevant features across modalities and how to fuse them. The rest of this section first reviews the related works on multimodal fusion and then provides an overview of our proposed method.

### 1.1. Multimodal Fusion

Multimodal fusion, while very promising, poses new challenges. There are multiple ways to fuse the data and stages of encoding where to fuse. The effectiveness of the strategy varies depending on data modalities and tasks. One of the key factors is the similarity between the modalities. Highly heterogeneous data, such as imaging, genetics, and clinical data, might not be immediately fused. Their difference in type, signal-to-noise ratio, and dimensionality makes it very challenging to combine them without first projecting them into a similar space. The relationship between input and output is equally important to consider when fusing data. For example, clinical assessments reflect the direct impacts of the disease, while genetic data describe the building blocks of cells. The phenotype-related information available in clinical assessments requires considerably less processing than what might be required for genomic data to find the connection with a disease.

**Figure 1:**
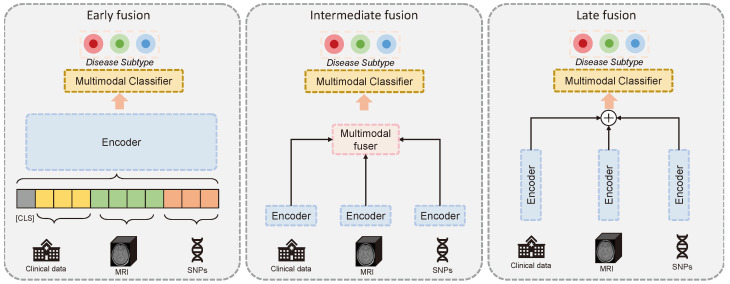
The three main multimodal fusion strategies, early, intermediate, and late fusion, for deep learning methods.

As illustrated in Figure 1, the existing multimodal fusion strategies can be grouped into three main categories [27], namely, early, intermediate (also called joint), and late fusion. It is essential to choose the right approach based on the task at hand and the data used. Early-stage fusion has shown very promising results in recent vision–language models [28,29], while late-fusion strategies have traditionally been very effective in aggregating machine learning model decisions. Early- and late-stage fusion strategies, while effective for certain tasks, are not ideal for AD subtyping using multimodal data. Early-stage fusion struggles with dealing with highly heterogeneous and differently biologically related data such as genetics, imaging, and clinical data. These require further encoding to first learn highly informative representations for every modality and condense them into a similar latent space. While late-stage fusion strategies can be very effective at aggregating the decisions based on each modality, they cannot learn the feature relationships across modalities, severely limiting the usefulness of the model. The intermediate fusion approach tackles both challenges by first learning the crucial patterns associated with each modality. In the next stage, it uses the condensed patterns from each modality to learn the cross-modal relationships. This enables a more harmonious fusion of the heterogeneous modalities.

### 1.2. Overview and Contributions

Despite the potential advantages of multimodal approaches, several technical challenges exist to effectively leverage the key data of each modality. The high heterogeneity of the data modalities for AD subtyping and the explicit learning of the cross-modal interactions need to be addressed. Previous approaches in related fields have proposed dual co-attention mechanisms to explicitly learn the cross-modal feature interaction and joint data representations [30]. While they have shown very promising results, these have yet to be explored for the progression-specific subtyping of neurodegenerative disease and its corresponding modalities. Moreover, in AD and related disorder subtyping, three critical but highly heterogeneous modalities are needed to be fused, i.e., imaging, genetics, and clinical data. Therefore, in this paper, a tri-modal co-attention (Tri-COAT) framework is proposed that can explicitly learn the interactions between the multimodal features towards the task of classifying subtypes. While deep learning models for disease assessment promise improved accuracy, they remain limited in the interpretability of the results. This is a major entry barrier for the inclusion of deep learning models in the medical field.

Our contributions in this paper are twofold in both the application and technique. First, our framework incorporates features of three early-stage biomarker modalities and provides a cutting-edge approach to the progression-specific subtyping of early neurodegenerative diseases. Second, regarding the technical innovation, our new tri-modal co-attention framework can efficiently and explicitly learn the interactions between highly heterogeneous modalities, encode the information into a joint representation, and provide explainability to the cross-modal interactions. The proposed Tri-COAT achieved state-of-the-art performance on the landmark Alzheimer’s Disease Neuroimaging Initiative (ADNI) dataset [31] and provided key insights into the biological pathways leading to neurodegenerative disease development.

The rest of the paper is organized as follows. Section 2 presents the methods with details of each component of Tri-COAT. Next, in Section 3, the dataset and experimental design are described. In Section 4, the results are presented and discussed. Finally, in Section 5, conclusions are drawn, and future directions are presented.

## 2. Method

**Figure 2:**
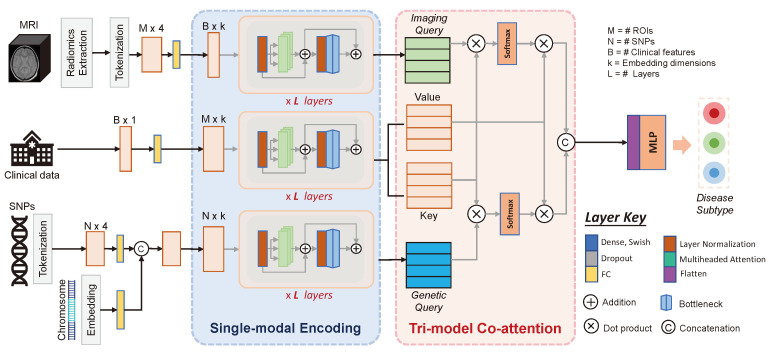
Illustration of the proposed framework for AD subtyping consisting of two main sections: single-modality encoding and tri-modal attention with joint encoding.

Our proposed framework can be divided into two main parts. As seen in Figure 2, single-modality encoders are first built using transformer modules to learn feature representations for each modality. Second, the Tri-COAT mechanism explicitly learns the critical cross-modal feature relationships and uses them to weigh the feature representation. The jointly learned representation is processed through a multilayer perceptron (MLP) for disease subtype classification.

### 2.1. Single-Modality Encoding

Three branches encode each modality individually. Each branch is comprised of a transformer encoder with several transformer layers. This is inspired by previous works in which transformer models have been proposed for imaging-derived connectomes [32] and genotype data [33]. Each branch learns representations of a modality to later combine them into a joint representation through the Tri-COAT mechanism.

*Imaging modality*. The imaging feature encoder branch uses MRI-derived quantitative traits as input. These quantitative traits are derived from T1-weighted MRI scans. The scans are first segmented based on the FreeSurfer atlas for cross-sectional processing. Next, for each reconstructed region of interest (ROI), the cortical region, cortical volume, thickness average, thickness standard deviation, and surface area are calculated. Further details are described in Section 3.1. The imaging traits are then used to build tokens, where each token represents an ROI in the brain and is comprised of four imaging-derived traits (cortical thickness average, cortical thickness standard deviation, surface area, and volume from cortical parcellation). Let $X_{I}\in R^{M\times 4}$ represent the imaging input to the proposed model, where M=72 and is the number of ROIs. Then, the token dimensions are expanded through a fully connected layer to match the model dimensions *k*. The imaging tokenization allows the building of an initial representation for each ROI rather than each trait, leading to a smaller number of input features and a more biologically informative and interpretable input.

*Genetic modality.* The genotype branch has as input single nucleotide polymorphism (SNP) data. After quality control and preprocessing of the genotype data as described in Section 3.1, tokens for each SNP are built. Each token is composed of the allele dosage from the patient, the corresponding odds ratio and rare allele frequency obtained from the most recent AD GWAS study, and whether the SNP is within an intergenic region (regulatory region) as a binary label. Then, the token dimensions are expanded through a fully connected layer to k/2. Let $X_{\mathrm{SNP}}\in R^{N\times k/2}$ represent the genotype input to the model, where N=70 and is the number of SNPs filtered out (see Section 3.1 for details). Moreover, based on the chromosome for which each SNP is located in, an additional embedding for each SNP can be built. By including the chromosome embedding, location knowledge for each SNP can be incorporated. Using an embedding layer, an embedding for each chromosome can be obtained $X_{\mathrm{Chr}}\in R^{N\times k/2}$. Finally, $X_{\mathrm{SNP}}$ and $X_{\mathrm{Chr}}$ are concatenated to obtain the final genotype embedding $X_{G}\in R^{N\times k}$. Similarly to the imaging data encoding, the genetic tokenization allows the building of more informative input structures to the genetic encoder. By providing additional attributes for each SNP beyond the patient mutation status, the model can learn richer patterns of characteristics that relate each SNP.

*Clinical modality.* The clinical data are already very closely related to the outcome of interest and contain only few features; therefore, no further extensive tokenization is performed. As there is just one value per clinical assessment, the tokens are directly built with one dimension. Let $X_{C}\in R^{B\times 1}$ represent the clinical input to our model, where B=7 and is the number of clinical features. Next, the token dimensions are expanded through a fully connected layer to match the model dimensions *k*.

*Single-modality encoders.* After the tokenization of each modality, they are fed into independent transformer encoders with *L* layers. The full process of the *L*-th layer in our transformer encoder is formulated as follows:

(1) $$F_{l}^{\prime }=\mathrm{MHA}\left(\mathrm{LN}\left(F_{l-1}\right)\right)+F_{l-1},$$

and

(2) $$F_{l}=\mathrm{FF}\left(\mathrm{LN}\left(F_{l}^{\prime }\right)\right)+F_{l}^{\prime },$$

where LN(·) is the normalization layer [34] and MHA(·) is multihead self-attention [35].

### 2.2. Tri-Modal Co-Attention

After each transformer encoder has learned a new representation for each modality, these are then used to learn the cross-modal feature relationships to guide the co-attention process on the clinical branch. In other words, the imaging and genomic features are employed to modulate the clinical learning process by highlighting the key hidden features that share relationships across modalities. The intuition behind the proposed approach is that as the clinical data are most closely related to the disease phenotype, this branch will carry most of the necessary information to classify the patients. Nevertheless, the imaging and genomic data also provide valuable information. The idea is analogous to the clinical data being the subject and verb in a sentence while the imaging and genomic data are the adjectives and adverbs. These two elements enrich the representation of the health status of a patient, analogous to enriching a sentence for a fuller meaning.

Let $X_{Emb}\in R^{\{M,N,B\}\times k}$ represent the learned representation of a given single-modality encoder. These become query matrices for the genetics $Q_{G}$ and imaging $Q_{I}$ data, and key $K_{C}$, value $V_{C}$ matrices for the clinical data. Following an attention mechanism structure, the co-attention between two modalities is computed as follows:

(3) $$\mathrm{CoAttn}\left(\left\{G,I\right\},C\right)=\mathrm{softmax}\left(\frac{Q_{\left\{G,I\right\}}K_{C}^{T}}{\sqrt{d_{k}}}\right)V_{C}^{T}.$$

Next, the resulting co-attention filtered value matrices are concatenated to obtain a final joint representation. This joint representation is then flattened and used to classify the patients into the clusters through an MLP.

## 3. Materials and Experiments

### 3.1. Dataset

**Figure 3:**
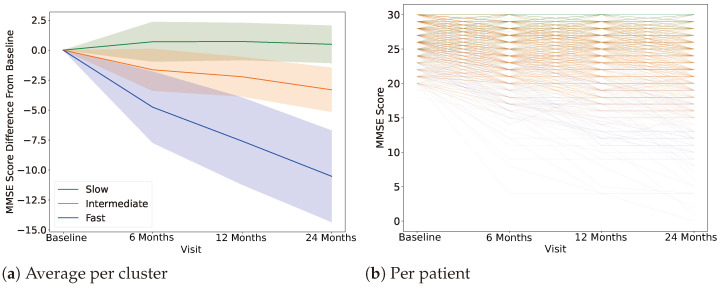
AD progression-specific subtype clusters based on a decrease in the MMSE at each visit. (**a**) Each line represents the average score across patients for each cluster, and the shadow represents one standard deviation. (**b**) Individual lines per patient are plotted.

The Alzheimer’s Disease Neuroimaging Initiative (ADNI) [31] database is a landmark dataset for the advancement of our understanding of Alzheimer’s disease. ADNI [31] is composed of a wide range of data modalities including MRI and PET images, genetics, cognitive tests, CSF, and blood biomarkers. Longitudinal data for all subjects were selected for up to two years of progression after disease onset due to the very high missingness rate (percentage of data points missing across patients for a given time point) present for time points after the two years. Subjects were clustered using k-means into three main groups based on their Mini-Mental State Examination (MMSE) scores as can be seen in Figure 3. These clusters match the cognitive decline rate of patients over time. The MMSE score at each visit (baseline, 6 months, 12 months, and 24 months) was used to determine the cognitive decline for each patient. As each patient may have a different starting level at baseline, the baseline measurement is subtracted from each of the following time points; thus, all patients start at 0. Then, using k-means clustering, using k=3, slow, intermediate, and fast cognitive decline groups are defined. As seen in Table 1, the raw average MMSE score at baseline is comparable across all groups with a steep decrease for the fast and intermediate groups at 24 months. The slow or otherwise stable group MMSE score at 24 months is comparable to the one at the initial stage. On the other hand, all three groups are age matched. Similarly, sex distributions across groups is maintained, with male subjects representing approximately 60% of the subjects for each group.

*Data preprocessing*: The data for the imaging and genotype modalities were processed previously by the tokenization process following best practices for the corresponding modality as described below.

*Imaging*: The FreeSurfer image analysis suite [36] was used to conduct cortical reconstruction and volumetric segmentation. T1-weighted MRI scans were segmented based on the FreeSurfer atlas for cross-sectional processing, enabling group comparison at a specific time point [37]. For each reconstructed cortical region, cortical volume, thickness average, thickness standard deviation, and surface area measurements were labeled by the 2010 Desikan-Killany atlas. The UCSF ADNI team conducted this process [38].

*Genotype*: The genotype variants were filtered using the intersection between the List of AD Loci and Genes with Genetic Evidence Compiled by the ADSP Gene Verification Committee and the most recent genome-wide association study (GWAS) on AD [39]. The odds ratios, rare allele frequency, and intergenic region binary trait were obtained from the most recent GWAS study with accession number (GCST90027158), accessed through the GWAS catalog [40]. Furthermore, the genotype variants were processed for sample and variant quality controls using PLINK1.9 [41].

*Clinical*: The clinical assessment features corresponded to seven different cognitive metrics available through ADNI [31]. These were Logical Memory-Delayed Recall (LDELTOTAL), Digit Symbol Substitution (DIGITSCOR), Trail Making Test B (TRABSCOR), and Rey Auditory Verbal Learning Test (RAVLT) scores: immediate, learning, forgetting, and percent forgetting. Values for the imaging and clinical modalities were normalized for each training set before they were used as inputs to the network.

### 3.2. Experimental Design

All models underwent training and evaluation using a 10-fold stratified cross-testing approach. Initially, the entire dataset was divided into 10 folds, with one fold reserved for testing and the remaining nine for training. Subsequently, this training set was further divided into 5 folds for a 5-fold stratified cross-validation process for hyperparameter tuning. This robust framework was designed to prevent any data leakage. The optimal hyperparameters were determined during each experimental run by selecting the best-performing model based on the validation set. Each of the 10 test sets was evaluated 5 times, using hyperparameters determined by the validation sets, resulting in a total of 50 evaluations for each method. Predictions were evaluated using the area under the receiver operating curve (AUROC). A one-vs-one strategy was employed where the average AUROC of all possible pairwise combinations of classes was computed for a balanced metric. This was implemented using Sci-Kit Learn API [42], which implements the method described in [43]. The mean AUROC and standard deviation across all 50 runs are reported for each method in Table 2. The model was compared against the stage-wise deep learning intermediate fusion model introduced in [44] and several well-established traditional ML models—random forest (RF) and support vector machine (SVM) with radial-basis function (RBF) kernel. Similarly, each of the branches of the model was used as comparison using a series of transformer encoder layers and MLP head for classification.

Tri-COAT consists of four transformer layers for each of the single-modality encoders, with four attention heads per transformer layer. The tri-model co-attention process is done in a single-head attention mechanism. The classifying MLP has one hidden layer with 256 units. An embedding dimension of k=256 was used for all modalities. The model dimensions for the single-modality encoders were kept at 256 throughout, as this combination achieved the best results on the validation set. The final MLP had the concatenated class tokens resulting from the tri-modal co-attention module and computed the output logits for each one of the three possible classes. Adam was used to optimize both the Tri-COAT and stage-wise MLP models using learning rates of 0.0001 for Tri-COAT and 0.0001 for the stage-wise fusion benchmarking model. All deep learning models were trained using cross-entropy loss. All deep learning models were implemented using PyTorch, while the RF and SVM models were implemented using scikit-learn. All deep learning models were trained for 100 epochs, and the best checkpoint, meaning the epoch with the highest AUROC for the validation set, was selected for model evaluation. The stage-wise deep learning fusion model had dimensions of 64 units for the single-modality layers, 32 for the second-stage, and 16 for the final stage. The model dimensions were selected following the described hyperparameters in [44]. The SVM used an RBF kernel and a regularization parameter C of 1. The random forest used gini as its criterion for leaf splitting, 100 trees, and no maximum depth.

## 4. Results and Discussion

### 4.1. Clustering, Label Definition

Following the literature, the number of clusters was set to three main groups [45,46,47]. The MMSE score was used as an indicator of mental decline. Based on the speed of the progression of the mental decline over a period of two years, three groups were defined, i.e., the fast-, intermediate-, and slow-progressing subtypes. K-means clustering was used to assign each subject to one of the groups. Through this process, labels were defined for all subjects. Only baseline data were used as input to Tri-COAT and all competing models. Based on the baseline data, Tri-COAT was able to effectively classify the subjects into their corresponding progression-specific subtypes.

### 4.2. AD Progression-Specific Subtype Classification

As seen in Table 2, Tri-COAT outperformed the single-modality ablations and baseline models, achieving an average AUROC of 0.734 ± 0.076 across all test sets in the 10-fold cross-testing framework. For the single-modality ablation studies, each modality was used independently to classify the AD subtype. For Tri-COAT, a single-modality transformer encoder backbone and MLP head were used. For the stage-wise fusion model, it was adapted to MLPs using the same number of hidden layers as the first plus last stage of the multimodal version. For SVM and RF, no variations were required. Each modality was evaluated using the same 10-fold cross-testing–5-fold-cross-validation hyperparameter tuning framework. Moreover, the single-modality ablation models outperformed their baseline counterparts for the clinical and genetics modalities. In contrast, the baselines achieved better performances for the imaging-derived traits. For the three modalities, the clinical modality achieved the best classification AUROC, followed by the imaging and genetics modalities. This is expected, as biologically, the same order follows for the closest relation between the observed phenotype and the mechanisms behind it. Clinical (cognitive) assessments are the closest to the MMSE metric, followed by imaging (changes in the brain morphology), which is directly related to the observed phenotype and genetics being the farthest apart from the expressed symptoms. Both the comparative models and Tri-COAT achieve higher performances in their multimodal configuration compared to single modalities, agreeing with previous literature regarding multimodal approaches for classification of AD and related disorders.

Furthermore, as seen in Table 3, Tri-COAT outperformed variations of itself using alternative fusion strategies. The early fusion model considerably underperforms achieving an AUROC of 0.571 ± 0.053 because of its limited capabilities to simultaneously encode highly heterogeneous data with distinct biological-level relationships to the outcome. Similarly, the late-stage fusion model underperforms, as it is limited to joining the predictions from each branch and cannot learn the relationships between the different modalities in the latent space.

### 4.3. Biomarker Associations Learned by Co-Attention

**Figure 4:**
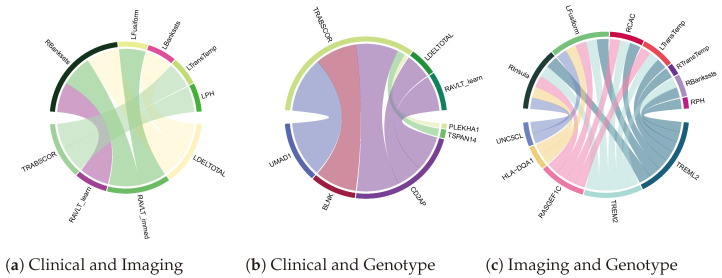
Cross-modal associations of key AD biomarkers visualized from the learned co-attention.

One of the key advantages of Tri-COAT compared to the baseline models and traditional deep learning approaches is the ability to learn insights into the cross-modal feature associations. In order to explore the learned relationships, the model with the highest test AUROC from the evaluation framework was selected for attention visualization in which the learned attention scores were averaged across all test subjects. Chord plots were drawn using the circlize R library [48] to visualize the cross-modal attention. As seen in Figure 4, Tri-COAT identified key associations between the Trails making test B score (TRABSCORE) in the clinical–imaging and clinical–genetics associations. This score tests for the cognitive ability of the patient for working memory and secondarily task-switching ability [49]. The clinical literature shows a strong correlation between gyri structures—temporal gyrus and parahippocampal gyrus (LTransTemp, LPH)—and the TRABSCORE [50]. Similarly, for the clinical–genotype association, TRABSCORE was found to be associated with the CD2AP gene, which has been clinically identified as a driver for the AD hallmark—neurofibrillary tangles (NFT) in the temporal gyrus region [51]. This is a very exciting finding for our network, as it establishes a putative relationship between genetics (CD2AP gene), brain ROIs (temporal gyrus), and clinical symptoms (TRABSCORE).

## 5. Conclusions

AD is the most prevalent neurodegenerative disease, and all current treatments are limited to slowing disease progression. Therefore, early diagnosis is essential. Furthermore, there are multiple subtypes with different rates of cognitive decline. In order to move closer to personalized medicine, it is essential to have a better understanding of the heterogeneity surrounding the development of this disease. However, early subtyping is a very challenging task. Our proposed model, Tri-COAT, was able to effectively classify AD patients into three main progression-specific subtypes using prodromal factors measured at baseline.

Moreover, the model was able to identify multiple putative cross-modal biomarker networks. The putative biomarkers provide enhanced interpretability for Tri-COAT and shed light on possible exciting therapeutic targets. Nevertheless, the generalizability of applying the learned features to other datasets remains to be tested.

The future directions are very exciting, as Tri-COAT could be extended to other heterogeneous neurodegenerative diseases such as Parkinson’s disease. Moreover, as shown in this work, multimodal approaches achieved the best results. A promising future direction is to incorporate further modalities such as PET imaging and transcriptomic data. PET imaging could provide further clarity towards the accumulation of fluid biomarkers and their impact towards neurodegeneration. Similarly, transcriptomic data could provide an intermediate biological step between the genotype and brain endophenotypes. These could lead to an enhanced understanding of the underlying mechanisms and provide further therapeutic targets.

## Figures and Tables

**Table 1:** Subject data distribution reported as the mean ± standard deviation for the MMSE and age and as counts per category for the number of participants and sex.

	Slow	Intermediate	Fast
Participants	177	302	15
MMSE (Baseline)	27.35 ± 2.51	27.66 ± 1.86	24.93 ± 3.55
MMSE (24 months)	28.15± 2.15	23.86 ± 3.68	15.9 ± 4.84
Age	73.26 ± 7.82	72.44 ± 7.55	71.22 ± 3.922
Sex	M: 102 F: 75	M: 185 F: 117	M: 9 F: 6

**Table 2:** Mean AUROC ± SD of 10-fold cross-testing results. The proposed model significantly outperformed all the baseline models. The statistical significance was evaluated by paired *t*-test with α=0.005, except for the entry where α=0.05 (shown in italics).

Method	Full	Imaging	Genetics	Clinical
SVM	*0.705 ± 0.036*	0.669 ± 0.060	0.525 ± 0.034	0.639 ± 0.078
RF	0.684 ± 0.048	0.677 ± 0.052	0.505 ± 0.031	0.659 ± 0.087
Stage-wise fusion	0.641 ± 0.017	0.557 ± 0.096	0.562 ± 0.078	0.655 ± 0.057
Tri-COAT	**0.734 ± 0.076**	0.648 ± 0.056	0.539 ± 0.084	0.697 ± 0.063

**Table 3:** Mean AUROC ± SD of 10-fold cross-testing results. Tri-COAT significantly outperformed early and late fusion variants. The statistical significance was evaluated by paired *t*-test with α=0.005, except for the entry where α=0.05 (shown in italics).

Method	AUROC
Early	0.571 ± 0.053
Late	*0.604 ± 0.048*
Tri-COAT	**0.734 ± 0.076**

## Data Availability

All data used in the preparation of this article were obtained from the Alzheimer’s Disease Neuroimaging Initiative (ADNI) database (adni.loni.usc.edu). The ADNI was launched in 2003 as a public-private partnership, led by Principal Investigator Michael W. Weiner, MD. The primary goal of ADNI has been to test whether serial magnetic resonance imaging (MRI), positron emission tomography (PET), other biological markers, and clinical and neuropsychological assessment can be combined to measure the progression of mild cognitive impairment (MCI) and early Alzheimer’s disease (AD).
